# Expression of Concern to: The α7 nicotine acetylcholine receptor agonist GTS-21 improves bacterial clearance in mice by restoring hyperoxia-compromised macrophage function

**DOI:** 10.1186/s10020-020-0143-9

**Published:** 2020-02-03

**Authors:** Ravikumar A. Sitapara, Daniel J. Antoine, Lokesh Sharma, Vivek S. Patel, Charles R. Ashby, Samir Gorasiya, Huan Yang, Michelle Zur, Lin L. Mantell

**Affiliations:** 1 Department of Pharmaceutical Sciences, College of Pharmacy and Health Sciences, St. John’s University, Queens, New York, USA; 2 Medical Research Council Centre for Drug Safety Science, Department of Molecular and Clinical Pharmacology, University of Liverpool, Liverpool, UK; 3 Laboratory of Biomedical Science, Feinstein Institute for Medical Research, North Shore-LIJ Health System, Manhasset, New York, USA; 4 Center for Inflammation and Immunology, Feinstein Institute for Medical Research, North Shore-LIJ Health System, Manhasset, New York, USA; 5 Center for Heart and Lung Research, Feinstein Institute for Medical Research, North Shore-LIJ Health System, Manhasset, New York, USA

## Abstract

The Editors-in-Chief would like to alert readers that this article (Sitapara et al. 2014) is part of an investigation being conducted by the journal following the conclusions of an institutional enquiry at the University of Liverpool with respect to the quantitative mass spectrometry-generated results regarding acetylated and redox-modified HMGB1.

**Expression of Concern to: Mol Med**

**https://doi.org/10.2119/molmed.2013.00086**

The Editors-in-Chief would like to alert readers that this article (Sitapara et al. 2014) is part of an investigation being conducted by the journal following the conclusions of an institutional enquiry at the University of Liverpool with respect to the quantitative mass spectrometry-generated results regarding acetylated and redox-modified HMGB1. Appropriate editorial action will be taken once the investigation is concluded.

Lokesh Sharma, Charles R. Ashby Jr., Saamir Gorasiya, Huan Yang, and Lin L. Mantell agree to this editorial expression of concern.

Ravikumar A. Sitapara, Vivek S. Patel, and Daniel J. Antoine have not responded to any correspondence from the editor/publisher about this editorial expression of concern.
